# Growth, Somatic Maturation, and Their Impact on Physical Health and Sports Performance: An Editorial

**DOI:** 10.3390/ijerph19031266

**Published:** 2022-01-24

**Authors:** Francesco Campa, Gianpiero Greco

**Affiliations:** 1 Department of Biomedical and Neuromotor Sciences, University of Bologna, 40126 Bologna, Italy; 2 Department of Basic Medical Sciences, Neuroscience and Sense Organs, University of Study of Bari, 70121 Bari, Italy; gianpierogreco.phd@yahoo.com

Over time, complex interactions and a nonlinear progression among a wide range of variables contribute to the improvement of physical health and of the elite level achievement in youth sport practitioners. Various elements, including technical skills, physical performance, environmental circumstances, and social conditioning, contribute to the development of these processes [1,2].

An influencing factor of growth and physical performance is somatic maturation [3,4]. The pubertal period is a critical time frame for skill acquisition and the development of performance in young people, in which suitable training strategies should be adopted to preserve the state of health while avoiding the risk of injury [5]. Athletes with similar chronological age competing in the same category levels can, in fact, show a difference in maturity status, and therefore in size, function, and body structure [6,7]. Physical and psychological differences related to maturity status and birthdate amongst athletes of the same selection year have been identified in a variety of sports and could be linked with the dropout of youth practitioners and a reduction in the talent pool [6].

Contemporary researchers have contributed to research on improving health and sports performance through the development of new measurement methods and training strategies in young athletes [8,9]. The aim of this Special Issue of *IJERPH* entitled Growth, Somatic Maturation, and Their Impact on Physical Health and Sports Performance [10] is to propose and evaluate new training strategies aimed at improving the health status and physical performance of young athletes while highlighting the relationship between somatic maturation, anthropometry features, education, and health-related factors via longitudinal and cross-sectional studies.

A total of 11 manuscripts are published here on different topics related to youth subjects and sports practice, as shown in Figure 1. Three papers are on physical performance aspects [11,12,13], five papers provide innovative findings in relation to anthropometry and body composition features [14,15,16,17,18], one paper examines the difficulties of running online physical education classes in the context of COVID-19 [19], and two are on the influence of training strategies on muscle strength and blood pressure [20,21].

Concerning physical and sports performance, Silva et al. [11] revealed that chronological age plays a determinant effect on vertical jump, linear sprint, and change of direction, revealing that older soccer players achieve better performance. On the contrary, playing position is not determined by age. Different player roles in youth basketball players were considered in the study of Ivanovic and co-workers [12]. The authors showed that (i) change of direction speed is the highest-ranked characteristic in basketball guards; (ii) jump performance is the highest-ranked feature in forwards; (iii) control of specific movements while dribbling the ball is the higher-ranked aspect in centers. Lastly, Ha et al. [13] demonstrated that neurocognitive function tests should not be used to predict lower extremity injuries in collegiate athletes.

Most of the papers published in this Special Issue focus on body composition, growth, and sports practice. In fact, body composition assessment is an important practice in sports management [22,23], given its numerous implications on the health and physical performance of youth subjects [24]. Obesity, child motor development, and physical fitness are influenced by socioeconomic status. In fact, Africa et al. [14] showed that in contrast to Western countries, children with lower socioeconomic status are leaner with a lower body fat and have better locomotor skills compared to their higher socioeconomic status peers in South Africa. Slankamenac et al. [16] provided specific somatotype profiles for Montenegrin karatekas, highlighting the peculiarities in body shape among different age and weight categories. Similarly, Penichet-Tomas et al. [17] published anthropometric references for male and female traditional rowers. The effect of age on body composition was also evaluated by Cattem and colleagues [18] in male and female athletes, who reported that subjects older than 13 years exhibited high fluid content and cell mass using qualitative and quantitative bioimpedance-based assessments. These data should be carefully considered in when developing training programs and talent selection procedures. In addition, Athayde et al. [15] reported that age at menarche and somatic growth are the primary indicators of physical performance in young female judo athletes.

Only one study examined the difficulties of running online physical education classes in the context of COVID-19 and used the findings to develop an efficient operation plan to address these difficulties [19]. The authors suggested that changes in strategic learning methods are needed to understand online physical education characteristics and thereby better communicate the value of physical education. They reported that it is necessary to cultivate teaching expertise through sharing online physical education classes, where collaboration among physical education teachers is central. In addition, the authors suggested that the evaluation processes should be less formal to encourage active student participation.

Two studies concerned resistance training practice and its effect on muscle strength and blood pressure in adolescents and young adults. Gullielm et al. [20] systematically reviewed and meta-analyzed the current evidence for the effects of resistance training on blood pressure in children and adolescents, concluding that this kind of exercise has no adverse effects on blood pressure and may positively affect it in youths. Lastly, Nunes et al. [21] suggested that biceps brachii muscle adaptations following a 10-week training program is almost identical regardless of whether peak torque emphasis was carried out in the final degrees or initial degrees of the range of motion in young adults.

In conclusion, as well as growth and development impacting children’s sporting experience and physical characteristics, sports practice can also impact children’s development and performance. This Special Issue reveals that the evolution of a healthy and successful athlete has a multifaceted nature and that several evaluation and training strategies are currently available to practitioners.

## Figures and Tables

**Figure 1:**
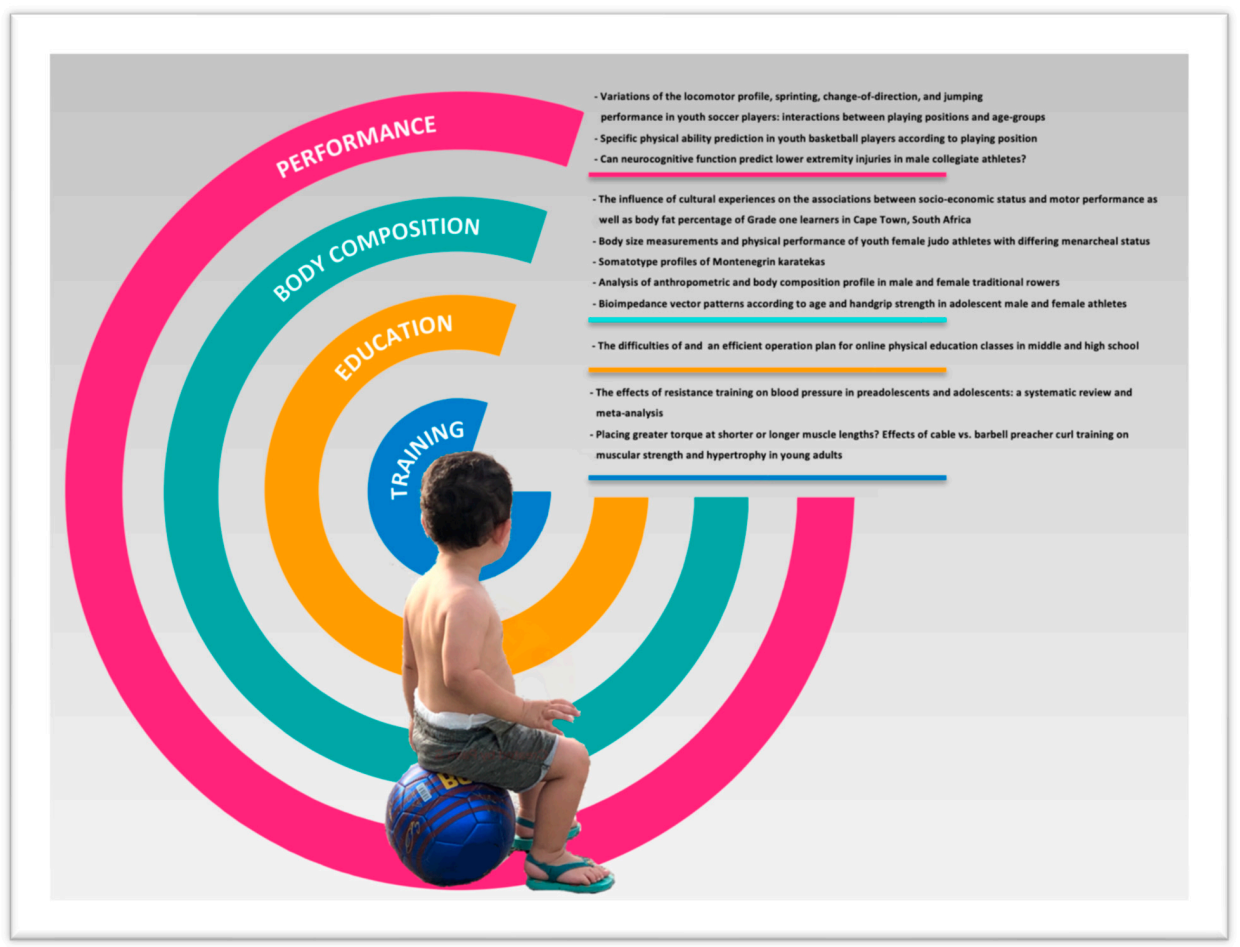
List of papers published in this Special Issue of IJERPH entitled Growth, Somatic Maturation, and Their Impact on Physical Health and Sports Performance [10].

## Data Availability

Not applicable.
